# Chemoprevention of intestinal tumorigenesis by the natural dietary flavonoid myricetin in *APC^Min/+^* mice

**DOI:** 10.18632/oncotarget.11108

**Published:** 2016-08-06

**Authors:** Ye Li, Shu-Xiang Cui, Shi-Yue Sun, Wen-Na Shi, Zhi-Yu Song, Shu-Qing Wang, Xin-Feng Yu, Zu-Hua Gao, Xian-Jun Qu

**Affiliations:** ^1^ Department of Pharmacology, School of Chemical Biology & Pharmaceutical Sciences, Capital Medical University, Beijing, China; ^2^ Beijing Key Laboratory of Environmental Toxicology, Department of Toxicology and Sanitary Chemistry, School of Public Health, Capital Medical University, Beijing, China; ^3^ Department of Pharmacology, Capital Medical University School of Basic Medical Sciences, Beijing, China; ^4^ Department of Pathology, McGill University, Montreal, Quebec, Canada

**Keywords:** myricetin, *APC^Min/+^* mouse model, intestinal adenomatous polyps, Wnt/β-catenin pathway, chronic inflammation

## Abstract

Myricetin is a natural dietary flavonoid compound. We evaluated the efficacy of myricetin against intestinal tumorigenesis in adenomatous polyposis coli multiple intestinal neoplasia (*APC^Min/+^*) mice. Myricetin was given orally once a day for 12 consecutive weeks. *APC^Min/+^* mice fed with myricetin developed fewer and smaller polyps without any adverse effects. Histopathological analysis showed a decreased number of dysplastic cells and degree of dysplasia in each polyp. Immunohistochemical and western blot analysis revealed that myricetin selectively inhibits cell proliferation and induces apoptosis in adenomatous polyps. The effects of myricetin were associated with a modulation the GSK-3β and Wnt/β-catenin pathways. ELISA analysis showed a reduced concentration of pro-inflammatory cytokines IL-6 and PGE2 in blood, which were elevated in *APC^Min/+^* mice. The effect of myricetin treatment was more prominent in the adenomatous polyps originating in the colon. Further studies showed that myricetin downregulates the phosphorylated p38 MAPK/Akt/mTOR signaling pathways, which may be the mechanisms for the inhibition of adenomatous polyps by myricetin. Taken together, our data show that myricetin inhibits intestinal tumorigenesis through a collection of biological activities. Given these results, we suggest that myricetin could be used preventatively to reduce the risk of developing colon cancers.

## INTRODUCTION

Colon cancer is the second leading cause of cancer death in Western countries and the third most common cancer in other parts of the world. Colon carcinogenesis is a multistep process with genetic and epigenetic alterations [1]. The loss of *APC* function plays a pivotal role in triggering these carcinogenetic events. *APC* is now recognized as a recessive tumor suppressor gene, and inactivation of both alleles is necessary for tumor formation. In the absence of functional *APC*, β-catenin-Wnt signaling is inappropriately and constitutively activated when β-catenin binds to its nuclear partners (members of the T-cell factor–lymphocyte enhancer factor family) [2]. Somatic mutations and deletions that inactivate both copies of *APC* are present in most sporadic colorectal adenomas and cancers. Patients with these genetic changes are 330 times more likely to develop colon cancer than their normal counterparts [3]. Most patients go undetected because symptoms are rare until the advanced stages of the disease. Clinical studies have indicated that half of the population develops at least one benign adenomatous colonic polyp, with about 3% of these progressing to colon cancer [1]. Thus, the presence of intestinal adenomatous polyps has been considered as a major precursor of colon cancer. Removing adenomatous polyps at this stage could prevent the development and progression to colon cancer. Fortunately, the transition from benign adenomatous polyps to advanced cancer takes several years, affording prime opportunities for early intervention. Chemoprevention of intestinal adenomatous polyposis has thus emerged as a pragmatic approach to reduce the risk of colon cancer.

Epidemiological and animal model studies have shown that the phytochemical ingredients of the diet play a major role in disease prevention due to their antioxidant properties, modulation of cell signaling pathways, modulation of gene expression and modulation of carcinogen metabolism [4–7]. Just as there are many dietary carcinogenic chemicals that are of environmental origin or generated through cooking, the diet also contains chemicals that are biologically active and proven to be effective against tumors in animal models and cell culture studies [8]. Nutritional prevention reduces occurrence of colon cancer by ~60% [9]. Flavonoids are bioactive compounds found in many foods such as fruit, vegetables, tea, chocolate and red wine [4]. A large body of evidence suggests that dietary flavonoids inhibit cancer cell proliferation, and promote apoptosis and cell cycle arrest [10–12]. One such potent citrus bioflavonoid is myricetin. Myricetin, 3,5,7,3′,4′,5′-hexahydroxyflavone, is a widespread naturally occurring flavonoid from the *Chrysobalanaceae* family, which can be found in most berries, fruits, vegetables and herbal medicines [13]. Previous reports have shown that myricetin possesses multiple biological activities, with antioxidant, anti-inflammatory, anti-carcinogenic and anti-proliferative effects [14]. However, these studies were mostly performed using *in vitro* assays with cancer cell lines. In this study, we aimed to evaluate the efficacy of myricetin on intestinal tumorigenesis using the *APC^Min/+^* mouse model. The *APC^Min/+^* mouse model is phenotypically similar to Familial Adenomatous Polyposis (FAP) in humans [15]. It is unique in that tumorigenesis develops spontaneously in the small intestine and the colon. The *APC^Min/+^* mouse is a powerful model for evaluating the effects of chemopreventive drugs against early-stage intestinal lesions. Myricetin-fed *APC^Min/+^* mice developed fewer and smaller intestinal adenomatous polyps than controls without any adverse effects. Myricetin selectively inhibited adenomatous cell proliferation, induced apoptosis, and reduced chronic inflammation in the small intestine and the colon. Given these biological properties, myricetin meets the basic requirements as a chemopreventive drug to reduce the risk of colon cancer.

## RESULTS

### Myricetin prevents intestinal adenomatous polyps in *APC^Min/+^* mice without any adverse effects

**Figure 1:**
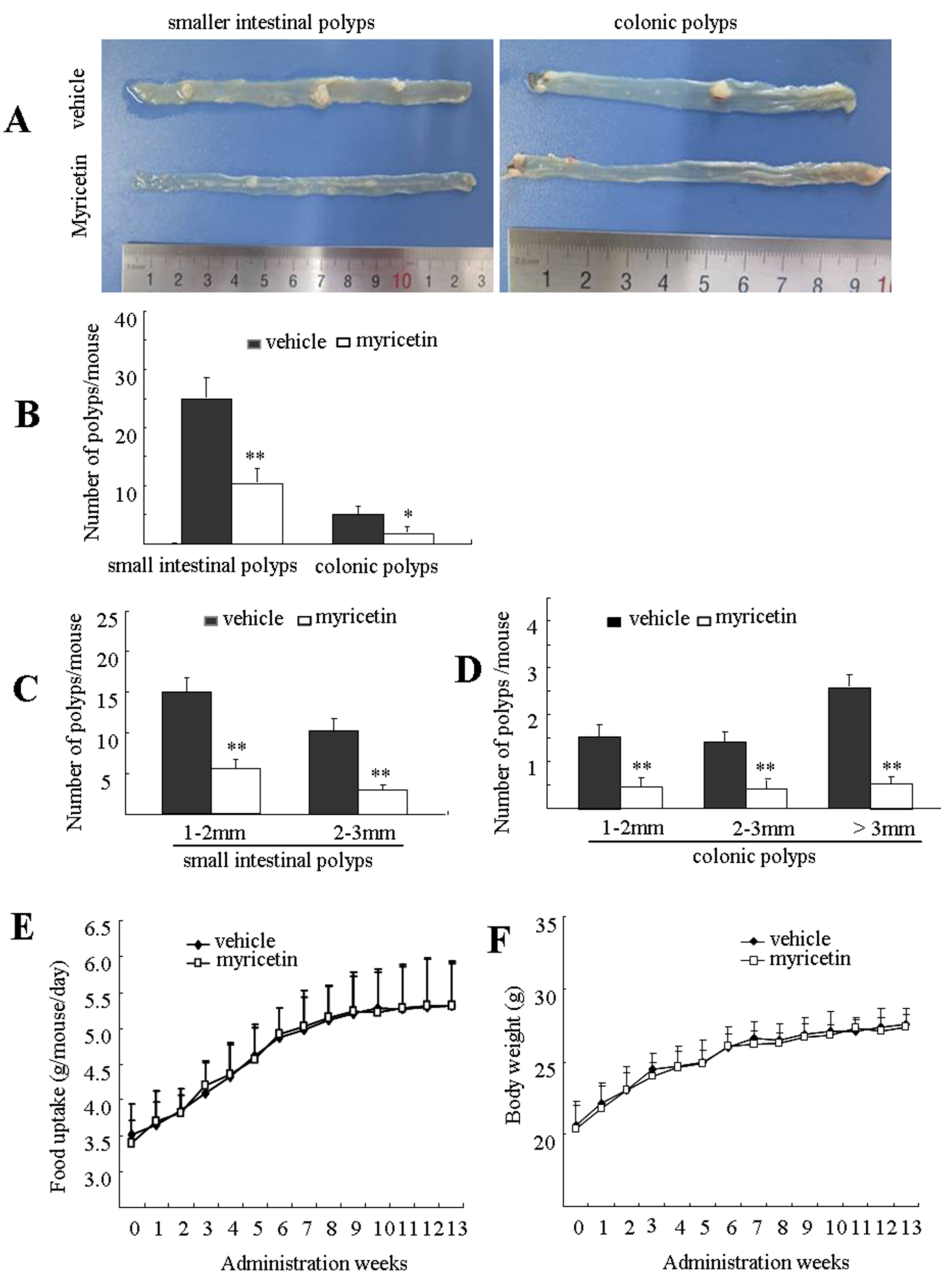
Myricetin prevents intestinal tumorigenesis in *APC^Min/+^* mice **A.** Representative pictures of distal small intestines and colonic intestines in vehicle control mice (top) and myricetin-treated mice (bottom). **B.** Number of polyps/mouse in the small intestine and colon in vehicle control mice (black bars) and myricetin treated mice (white bars). **C.** and **D.** Size distribution of polyps in small (C) and colonic intestines (D) in vehicle control mice (black) and myricetin-treated mice (white). **E.** and **F.** Myricetin treatment had no effect on food consumption (E) and body weight gain (F) in the *APC^Min/+^* mice. *, p < 0.05; **, p < 0.01 *vs.* vehicle control. Bars represent means ± S.D. of nine mice.

At 18 weeks of age, mice in the control group developed 25.2 and 3.5 polyps on average in the small intestine and colon, respectively (Figure 1A). Mice fed with myricetin developed fewer and smaller intestinal polyps. The total number of polyps in the myricetin-fed mice was significantly reduced by 58.9% (p < 0.05 *vs.* vehicle control) in small intestines and 71.8% (p < 0.01 *vs.* vehicle control) in colons (Figure 1B).

Size distribution analysis of adenomatous polyps showed differential effects of myricetin, depending upon polyp size and the intestinal segment where it was located. In the small intestine, myricetin reduced the total number of polyps that were 1 to 2 mm in size by 58.1% (p < 0.05 *vs.* vehicle control), and polyps that were 2 to 3 mm in size by 68.9% (Figure 1C, p < 0.01 *vs.* vehicle control). Myricetin reduced the total number of colonic polyps 1-2 mm, 2-3 mm, and > 3 mm in size by 70.8% (p < 0.01 *vs.* vehicle control), 72.4% (p < 0.01 *vs.* vehicle control) and 80.8% (p < 0.01 *vs.* vehicle control), respectively (Figure 1D).

During the term of treatment, myricetin-treated mice did not show any differences with vehicle treated mice in food consumption (Figure 1E), body weight (Figure 1F), nor any symptoms of toxicity in blood counts (Table 1).

**Table 1:** Peripheral blood element counts in *APC^Min/+^* mice

Groups	Number of mice (n)	Total WBC (×10^9^/L)	Neutrophils (×10^9^/L)	Lymphocytes (×10^9^/L)	Platelets (×10^9^/L)
**Vehicle**	9	6.71 ± 1.31	1.95 ± 0.87	5.43 ± 1.02	846.75 ± 106.02
**Myricetin**	9	6.62 ± 1.79	1.82 ± 1.07	5.49 ± 1.47	851.49 ± 121.47

*APC^Min/+^* mice were treated with myricetin by p.o. gavage daily for 12 consecutive weeks. Blood was drawn by exsanguination from the inferior vein. Blood elements were counted with an automated hematology analyzer.

### Myricetin inhibits the malignant progression of intestinal adenomatous polyps

**Figure 2:**
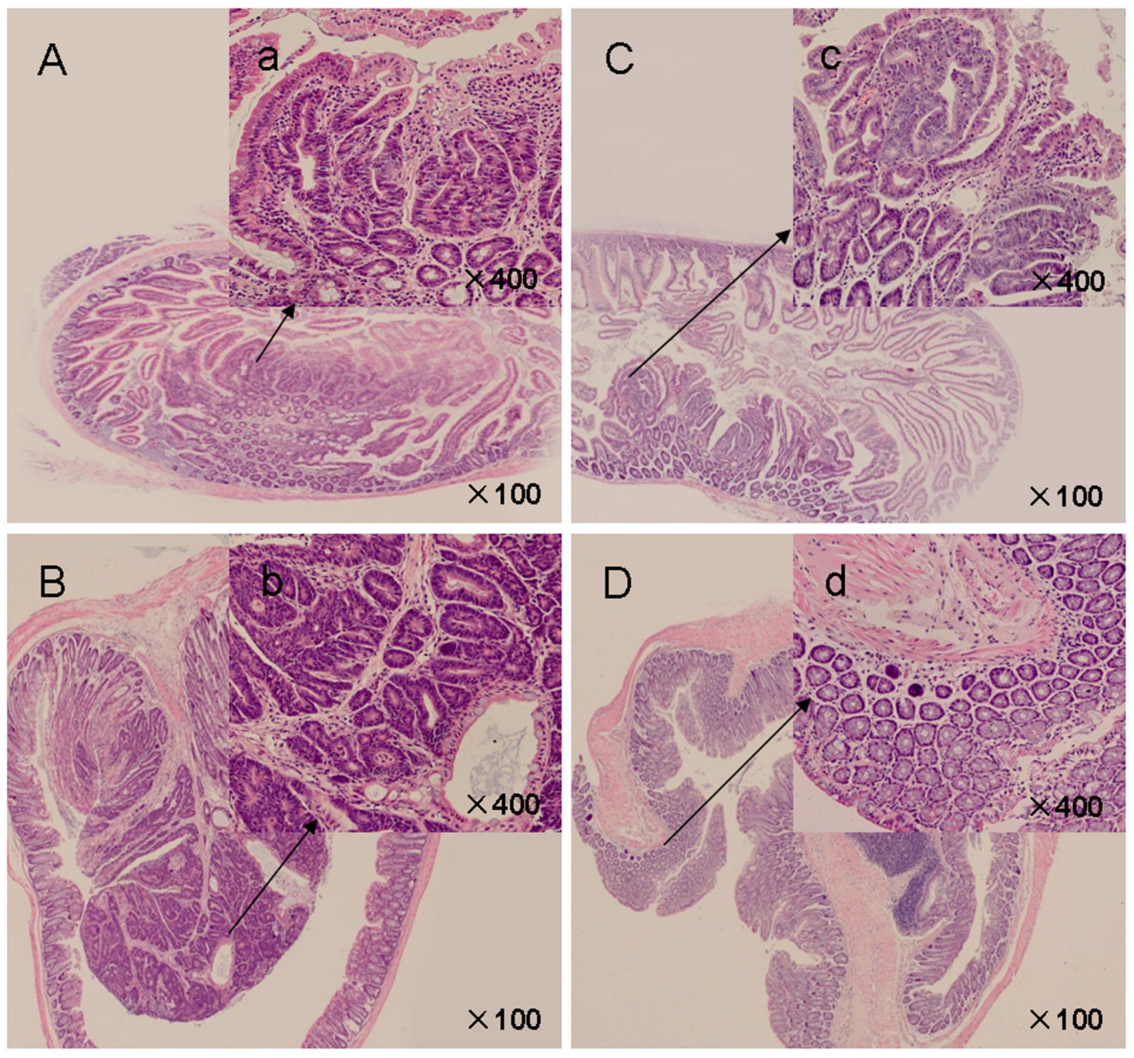
H&E-stained intestinal sections from *APC^Min/+^* mice **A.** Control mice presented crater-shaped adenomatous polyps in small intestines (×100). a' (inset): Adenomatous cells showed enlarged, hyperchromatic, elongated, and crowded dysplastic nuclei (×400). **B.** Advanced adenomatous polyps with focal high grade dysplasia in colonic polyps of vehicle control mice (×100). b' (inset): Crypt architecture appears complex, nuclei were pleomorphic with frequent mitoses and lack polarization (×400). **C.** Myricetin-treated small intestinal polyps (×100). c' (inset): Crypt architecture showed decreased dysplastic cells and degree of dysplasia (×400). **D.** Myricetin-treated colonic polyps (×100). d' (inset): Epithelium presented unremarkable nuclear structure (×400).

In *APC^Min/+^* mice, all intestinal polyps were histologically determined to be adenocarcinoma. At 18 weeks of age, the histology of small intestinal adenomatous polyps revealed nuclei of varying size and shape, with an increased nuclear-to-cytoplasmic ratio (Figure 2A–2a, inset). Larger adenomatous polyps in the colon demonstrated advanced adenomas with focal high grade dysplasia and/or intramucosal carcinomas with architectural distortion, lack of polarity, marked nuclear pleomorphism, and frequent abnormal mitosis (Figure 2B–2b, inset). Myricetin feeding inhibited the progression of intestinal adenomatous polyps, showing small adenomas. A reduction in the number of anaplastic cells and low grade dysplasia was observed in small intestinal (Figure 2C–2c, inset) and colonic adenomatous polyps (Figure 2D–2d, inset) of all the 9 myricetin-treated mice. High-grade dysplasia and intramucosal carcinoma were not observed, whereas interdigitated intestinal villi with a normal appearance were seen in the majority of the myricetin-treated small intestine and colon samples. The effect of myrecetin treatment was most prominent in larger sized polyps, especially those originating in the colon.

### Myricetin selectively inhibits proliferation and induces apoptosis in adenomatous polyps

**Figure 3:**
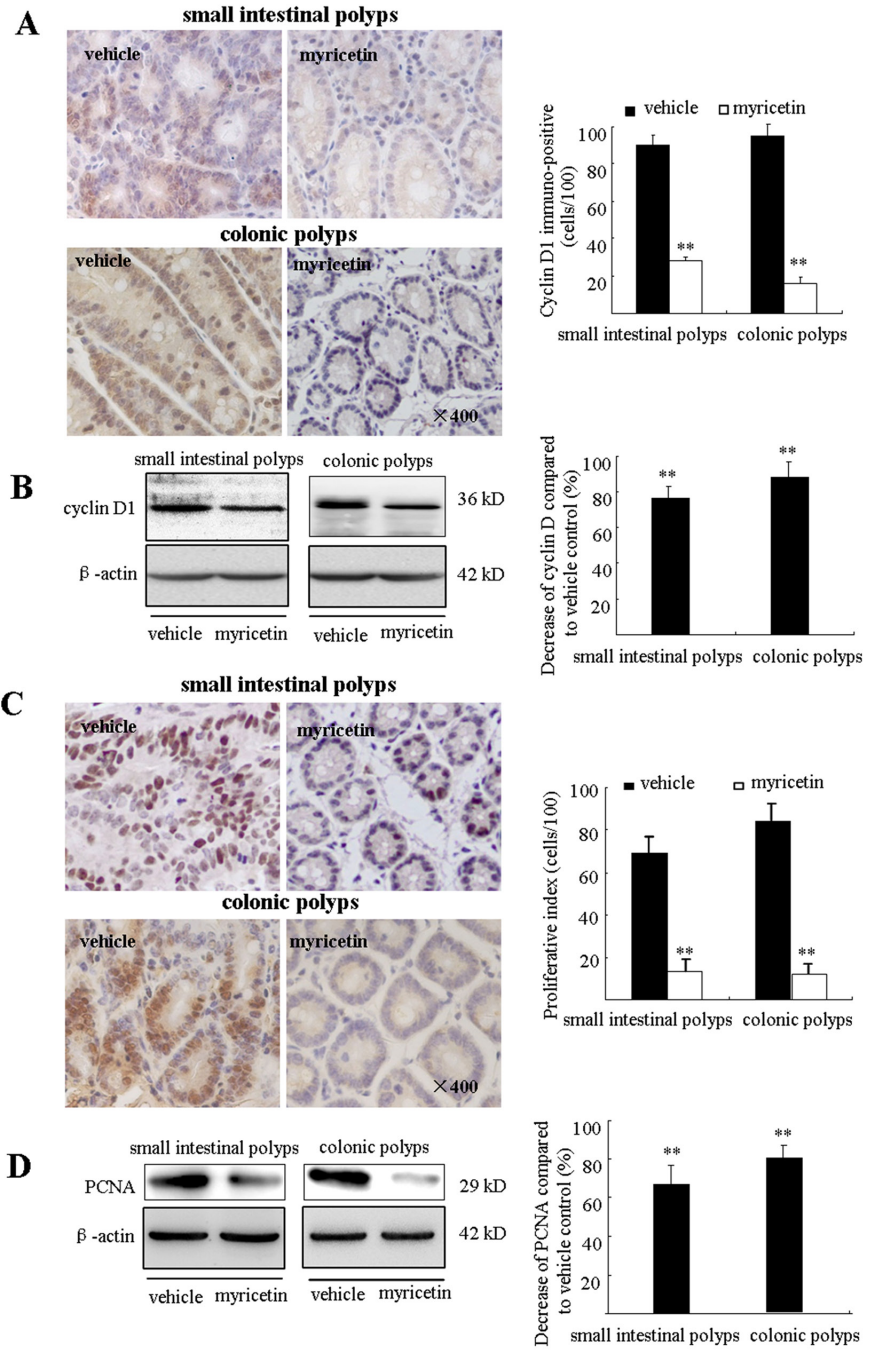
Myricetin reduces the expression of cyclin D1 and PCNA in intestinal adenomatous polyps **A.** (Left) Immunohistochemical staining shows numerous cyclin D1- positive cells in the small intestinal and colonic adenomatous polyps of vehicle control mice as compared to myricetin-treated mice (×400, local area). (Right) Bar graphs of cyclin D1-positive cell counts. **B.** (Left) Western blots of lysates from myricetin-treated mice show decreased cyclin D1 expression in the small intestinal and colonic adenomatous polyps as compared to vehicle controls. (Right) Band intensity as quantified by densitometry and normalized to β-actin. **C.** (Left) Myricetin decreased PCNA-positive cells as determined by immunohistochemical staining (×400, local area). (Right) Bar graphs of PCNA-positive cell counts. **D.** Western blot analysis shows reduced PCNA expression in the small intestinal and colonic adenomatous polyps of myricetin-treated mice as compared to vehicle controls. (Right) Band intensity as quantified by densitometry and normalized to β-actin. Bars represent means ± S.D. of six mice. **, p < 0.01 *vs.* vehicle control. Experiments were performed in triplicate.

Excessive proliferation and insufficient apoptosis are often associated with the development and progression of colon cancers. Cyclin D1 and PCNA have been widely accepted as cell proliferation markers. Immunohistochemical (IHC) analysis showed high levels of cyclin D1 and PCNA expression in intestinal adenomatous polyps. Myricetin treatment resulted in a decrease of cyclin D1 stained cells by 72.1% (p < 0.01 *vs.* vehicle control) and 83.9% (p < 0.01 *vs.* vehicle control) in the small intestine and colon, respectively (Figure 3A). Western blot analysis of adenomatous polyps in myricetin-treated mice showed an inhibition of cyclin D1 in both the small intestinal and colonic polyps by 76.6% (p < 0.01), and 88.4% (p < 0.01), respectively (Figure 3B). IHC analysis revealed a 73.1% reduction of PCNA stained cells in small intestine polyps (p < 0.01 *vs.* vehicle control), and a 84.1% reduction of PCNA stained cells in colonic polyps (p < 0.01 *vs.* vehicle control) (Figure 3C). Western blot analysis showed that the expression of PCNA was reduced by 70.3% (p < 0.01 *vs.* vehicle control) and 79.9% (p < 0.01 *vs.* vehicle control), in small intestinal and colonic polyps, respectively (Figure 3D).

**Figure 4:**
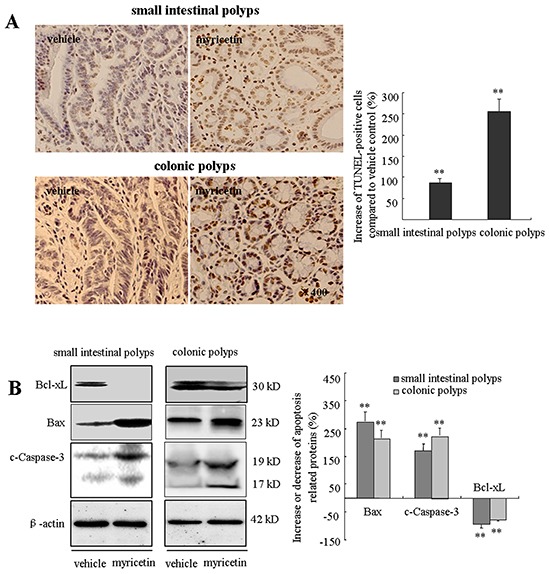
Myricetin induces apoptosis in the adenomatous polyps **A.** (Left) TUNEL stained adenomatous cells in small intestinal and colonic polyps in vehicle control and myricetin-treated mice (400×). (Right) TUNEL stained DAB-positive cells (with brown staining) were counted in five arbitrarily selected fields. Bars represent means ± S.D. of six mice. **, p < 0.01 *vs.* vehicle control. **B.** Western blot analysis of the expression of apoptotic proteins in small intestinal and colonic adenomatous polyps. Experiments were performed in triplicate. **, p < 0.01 *vs.* vehicle control.

Intestinal adenomatous polyps have insufficient apoptosis as compared to adjacent normal tissue. The apoptotic effect of myricetin was evaluated by TUNEL assay and Western blot analysis. In myricetin-fed mice, TUNEL-positive cells were significantly increased by 86.3% (p < 0.01 *vs.* vehicle control) and 255.2% (p < 0.01 *vs.* vehicle control) in small intestinal and colonic polyps (Figure 4A), respectively. Western blot analysis showed that myricetin increases the expression of proapoptotic Bax by 283.8% (p < 0.01 *vs.* vehicle control) and 191.6% (p < 0.01 *vs.* vehicle control); and the apoptotic executor c-Caspase-3 by 167.4% (p < 0.01 *vs.* vehicle control) and 226.1% (p < 0.01 *vs.* vehicle control), in small intestinal and colonic polyps, respectively. The expression of anti-apoptotic Bcl-xL was significantly reduced by 95.3% (p < 0.01 *vs.* vehicle control) in small intestinal polyps, and by 77.4% (p < 0.01 *vs.* vehicle control) in colonic polyps (Figure 4B).

### Myricetin modulates GSK-3β activity and β-catenin localization in adenomatous cells

**Figure 5:**
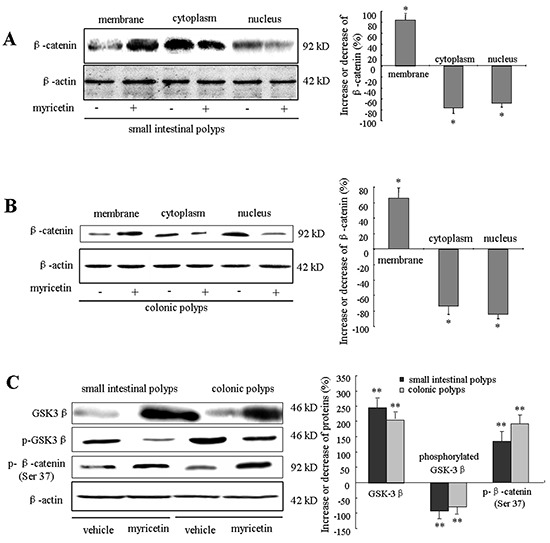
Myricetin modulates the localization of GSK-3β and β-catenin in intestinal adenomatous cells **A.** and **B.** Western blot analysis of β-catenin in the nuclear, cytoplasmic and membrane fractions of lysates from small intestinal (A) and colonic adenomatous polyps (B) in vehicle control and myricetin-treated mice. The percentage of decrease or increase of β-catenin was compared to vehicle control treated mice. **C.** Western blot analysis of GSK3β, p-GSK3β and p-β-catenin in intestinal adenomatous polyps. Experiments were performed in triplicate. Bar represents means ± S.D. of six mice. *, p < 0.05; **, p < 0.01 *vs.* vehicle control.

Myricetin treatment resulted in the inhibition of nuclear and cytoplasmic β-catenin expression, whereas the expression of membranous β-catenin was increased (Figure 5A and 5B). Western blotting of small intestinal polyps in myricetin treated mice showed that nuclear β-catenin was inhibited by 76.3% (*p* < 0.05 *vs.* vehicle control) and cytoplasmic β-catenin was inhibited by 68.4% (p < 0.05 *vs.* vehicle control), whereas membranous β-catenin increased by 83.5% (p < 0.01 *vs.* vehicle control) (Figure 5A). In colonic polyps, myricetin inhibited nuclear β-catenin by 84.6% (p < 0.01 *vs.* vehicle control) and cytoplasmic β-catenin by 75.4% (p < 0.01 *vs.* vehicle control). In contrast, membranous β-catenin was increased by 65.9% (p < 0.01 *vs.* vehicle control) in colonic polyps (Figure 5B).

To determine whether the effect of myricetin on β-catenin expression occurs through modulation of GSK-3β activity, we analyzed the expression of GSK-3β and phosphorylated β-catenin at Ser37 in the intestinal adenomatous cells. In *APC^Min/+^* mice, intestinal adenomatous polyps demonstrated low levels of active non-phosphorylated GSK-3β and high levels of phosphorylated GSK-3β (Figure 5C). Myricetin treatment resulted in an increase of active GSK-3β by 245.6% (p < 0.01 *vs.* vehicle control) and 203.8% (p < 0.01 *vs.* vehicle control), in small intestinal and colonic polyps, respectively. Myricetin treatment reduced the phosphorylated GSK-3β by 86.2% and 74.3%, in small intestinal and colonic polyps (p < 0.01 *vs.* vehicle control), respectively. Consequently, the expression of phosphorylated β-catenin at Ser37 was significantly increased in both small intestinal and colonic polyps following modulation of GSK-3β by myricetin (p < 0.01 *vs.* vehicle control).

### Myricetin inhibits chronic inflammation in the adenomatous polyps and blood

**Figure 6:**
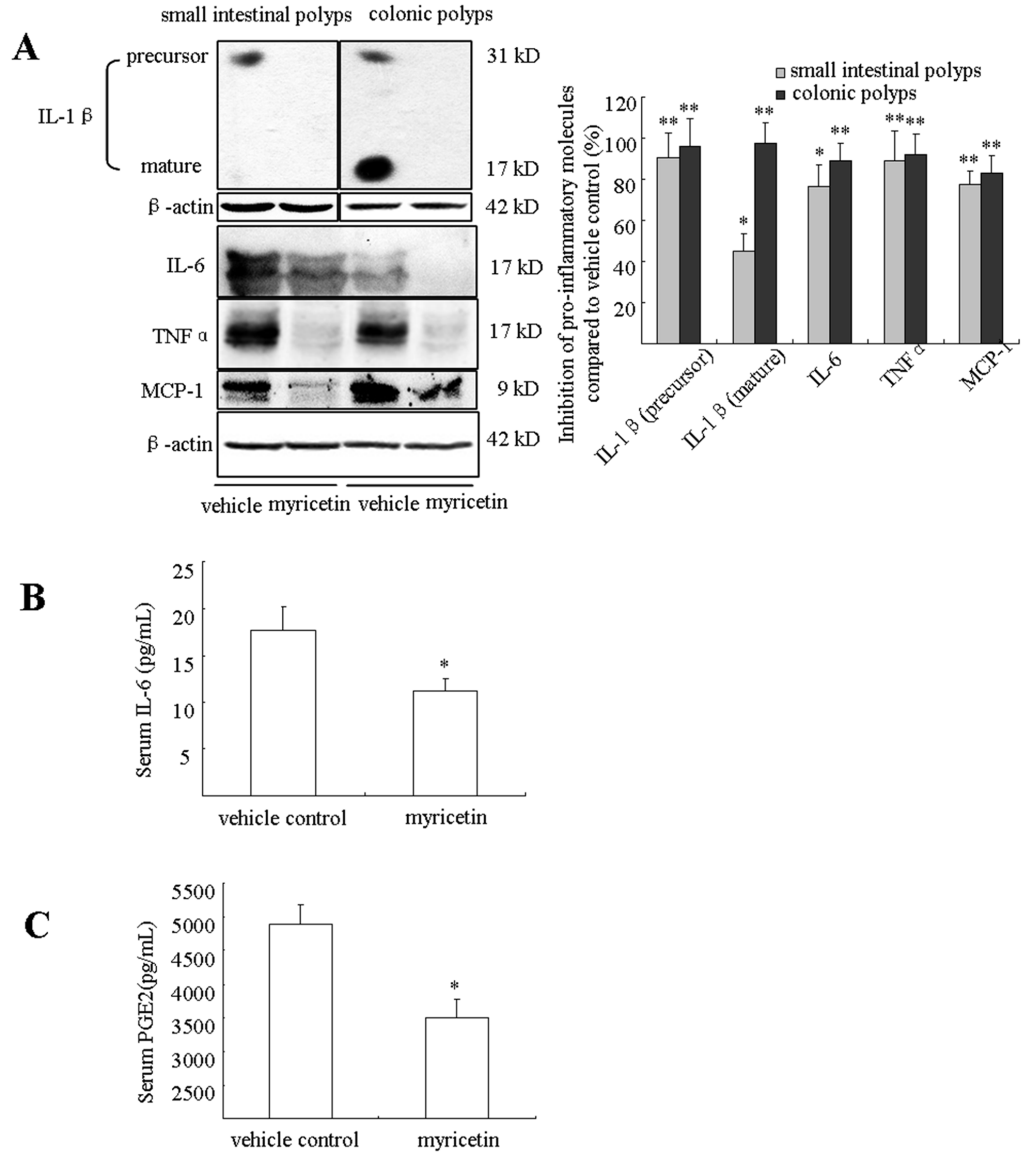
Myricetin reduces inflammatory cytokines in intestinal polyps and serum **A.** Western blots show a decrease of IL-1β, IL-6 and TNF-α in intestinal polyps in myricetin-treated mice. **B.** and **C.** Myricetin reduces serum concentrations of IL-6 (B) and PGE_2_ (C) as determined by ELISA. Bars represent means ± S.D. of six mice, performed in triplicate. *, p < 0.05; **, p < 0.05 *vs.* vehicle control mice.

Chronic inflammation plays important roles in colonic tumorigenesis, which is stimulated by a variety of pro-inflammatory molecules and growth factors. In the *APC^Min/+^* mouse model, the association of chronic inflammation with tumorigenesis has been described [16, 17]. We evaluated the effects of myricetin on pro-inflammatory cytokines IL-1β, IL-6, TNF-α and MCP-1 in the adenomatous polyps by western blot assay and concentration of PGE_2_ and IL-6 in blood by ELISA analysis. As shown in Figure 6A, both IL-1β precursor and mature IL-1β were strongly inhibited in small intestinal and colonic polypsas determined by western blotting assay. Further analysis showed that myricetin reduced the levels of IL-6 by 76.6% (p < 0.05 *vs.* vehicle control) and 89.2% (p < 0.01 *vs.* vehicle control); TNFα by 89.4% (p < 0.01 *vs.* vehicle control) and 91.9% (p < 0.01 *vs.* vehicle control); and MCP-1 by 77.2% (p < 0.01 *vs.* vehicle control) and 83.3% (p < 0.01 *vs.* vehicle control) in small intestinal and colonic polyps, respectively. We analyzed the concentration of IL-6 and PGE_2_ in blood by ELISA assay. In the vehicle-treated mice, the concentration of IL-6 was 17.68 ± 2.5 pg/mL. Myricetin treatment, however, significantly reduced the level of IL-6 to 11.2 ± 1.3 pg/mL (Figure 6B, p < 0.05 *vs.* vehicle control). The concentration of PGE_2_ was 4889.5 pg/mL ± 276.8 pg/mL in the vehicle-treated mice (Figure 6C), while myricetin treatment significantly reduced the concentration of PGE_2_ to 3505 pg/mL ± 263 pg/mL (p < 0.05 *vs.* vehicle control).

### Myricetin downregulates the phosphorylated p38 MAPK and Akt/mTOR signaling pathways

**Figure 7:**
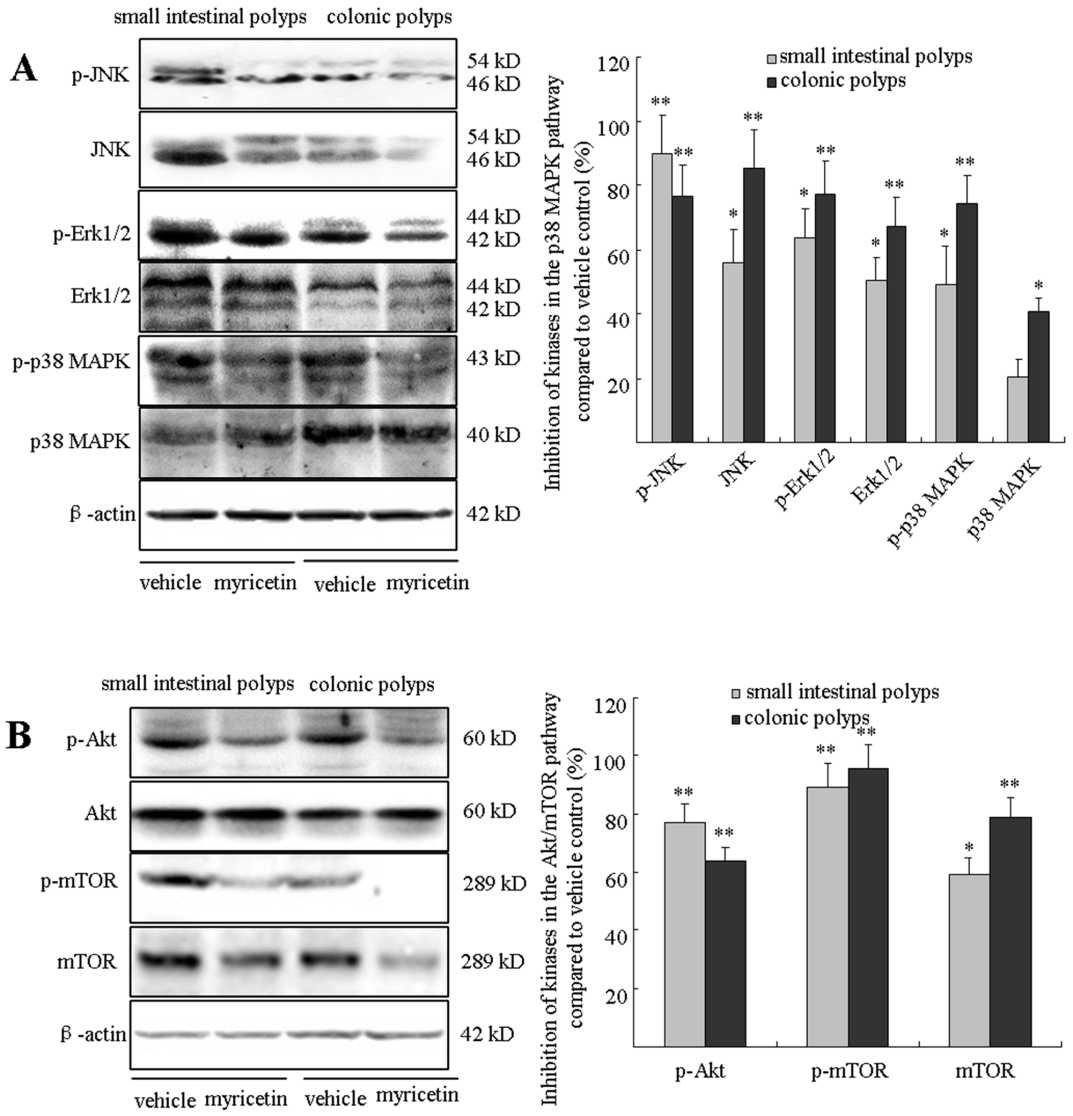
Myricetin downregulates the p38 MAPK and Akt/mTOR signaling pathways **A.** Western blot analysis of kinases in the p38 MAPK pathway in the adenomatous polyps. **B.** Myricetin inhibits the expression of kinases in the Akt/mTOR pathway in intestinal adenomatous polyps. Bars represent means ± S.D. of six mice, performed in triplicate. *, p < 0.05; **, p < 0.05 *vs.* vehicle control mice.

The p38 mitogen-activated protein kinase (MAPK) and protein kinase B (Akt)/mammalian target of rapamycin (mTOR) signaling pathways play crucial roles in sustaining ongoing colonic tumorigenesis. The *APC^Min/+^* mouse model is characterized by dysfunctional signaling of the phosphorylated p38 MAPK and Akt/mTOR pathways in adenomatous polyps [18]. The overexpression of these kinases in *APC^Min/+^* mice, as evaluated by Western blot analysis, was significantly reduced in the myricetin-treated adenomatous polyps. As shown in Figure 7A, myricetin inhibited the expression of p-JNK by 89.7 (p < 0.01 *vs.* vehicle control) and 76.6% (p < 0.01 *vs.* vehicle control); JNK by 56.2% (p < 0.05 *vs.* vehicle control) and 85.3% (p < 0.01 *vs.* vehicle control); p-Erk1/2 by 63.4% (p < 0.05 *vs.* vehicle control) and 77.2% (p < 0.01 *vs.* vehicle control); Erk1/2 by 50.2% (p < 0.05 *vs.* vehicle control) and 67.4% (p < 0.01 *vs.* vehicle control); p-p38 MAPK by 48.9% (p < 0.05 *vs.* vehicle control) and 74.3% (p < 0.01 *vs.* vehicle control); and p38 MAPK by 20.1% (p > 0.05 *vs.* vehicle control) and 40.3% (p < 0.05 *vs.* vehicle control) in small intestinal and colonic polyps, respectively. The effect of myricetin on the Akt/mTOR cascade is demonstrated in Figure 7B. Myricetin inhibited the expression of p-Akt by 76.9% (p < 0.01 *vs.* vehicle control) and 63.7% (p < 0.01 *vs.* vehicle control); p-mTOR by 89.2% (p < 0.01 *vs.* vehicle control) and 95.6% (p < 0.01 *vs.* vehicle control); mTOR by 59.1% (p < 0.05 *vs.* vehicle control) and 78.6% (p < 0.01 *vs.* vehicle control) in small intestinal and colonic polyps, respectively. Akt expression was not significantly changed in small intestinal and colonic polyps treated by myricetin (p > 0.05 *vs.* vehicle control).

## DISCUSSION

We have demonstrated that long term use of myricetin could prevent spontaneous intestinal tumorigenesis in *APC^Min/+^* mice. Myricetin-fed *APC^Min/+^* mice developed fewer and smaller adenomatous polyps in the intestines with a lower degree of cytological dysplasia. These effects of myricetin were associated with its anti-proliferative, apoptotic and anti-inflammatory biological activities. Myricetin strongly reduced proinflammatory cytokines of IL-1β, IL-6, TNF-α, and MCP-1 in adenomatous polyps and IL-6 and PGE_2_ in blood. Importantly, myricetin prevented intestinal tumorigenesis without any adverse effects. These properties of myricetin meet with the basic requirements for a chemopreventive agent. Thus, this natural dietary flavonoid could be used to reduce the risk of developing colon cancer.

Excessive growth and insufficient apoptosis are often associated with intestinal tumorigenesis [19, 20]. It has been widely accepted that early stage colon cancer is represented by a benign adenoma that could progress to adenocarcinoma *in situ* - tumors with high-grade dysplasia [15, 21]. The *APC^Min/+^* mouse model displays phenotypes reminiscent of these malignant processes in the development of colon cancer in humans [15]. Intestinal adenomatous polyps in *APC^Min/+^* mice exhibit dysplastic crypts surrounded by hyperplastic villi and crypts, resulting in “rose” shapes [22]. Intestinal adenomatous polyps have a higher mitotic index than adjacent normal tissue. Colonic tumors are peduncular, forming a spherical mass of dysplastic cells supported by a stromal stalk. Ulceration frequently occurs in adenomatous polyps greater than 3 mm in size [23]. In this study, myricetin significantly inhibited the formation of adenomatous polyps consistent with its anti-proliferative and pro-apoptotic activities. Histopathological analysis showed a reduction of dysplastic cells and a lower degree of dysplasia in the intestinal polyps. It is important to note that the apoptotic effect of myricetin was restricted to the intestinal adenomatous polyps. Long-term use of myricetin did not produce any adverse effects in the gastrointestinal system and blood element counts. These results suggest that myricetin could be used as a chemopreventive agent against intestinal tumorigenesis.

During the development of colonic tumorigenesis, *APC* is a canonical tumor suppressor. *APC* forms a “destruction complex” with Axin/Axin2 and GSK-3β that promotes the ubiquitination and subsequent proteasomal degradation of β-catenin [24]. Furthermore, GSK-3β destabilizes β-catenin by phosphorylating it at the sites of Ser33, Ser37 and Thr41 [25]. Mutations in these phosphorylation sites lead to the stabilization of β-catenin protein levels. Loss of *APC* function leads to the dysfunction of GSK-3β, and therefore results in an accumulation of β-catenin, which translocates to the nucleus and engages the Tcf/Lef transcription factor complex to activate the transcription of a large number of target genes, such as cyclin D1, PCNA, c-myc and CRDBP [26, 27]. In addition, serine/threonine kinase AKT is an important upstream regulator of GSK-3β. Activated Akt stimulates phosphorylated GSK-3β and further lead to an accumulation of β-catenin in the nucleus [28]. In *APC^Min/+^* mouse model, intestinal adenomatous polyps are characterized by low levels of active non-phosphorylated GSK-3β, high levels of phosphorylated GSK-3β and phosphorylated Akt and large accumulations of nuclear β-catenin [29]. In this study, myricetin inhibited phosphorylated Akt and modulated the expression of GSK-3β and β-catenin in the intestinal adenomatous polyps, showing that the level of active non-phosphorylated GSK-3β was increased and its non-functional phosphorylated form was decreased in the myricetin-treated group. Myricetin reduced nuclear and cytoplasmic β-catenin, and increased membranous β-catenin. Further analysis showed an increase of phosphorylated β-catenin at the site of Ser37. Therefore, we suggest that myricetin might inhibit intestinal adenomatous polyps through modulation of the GSK-3β and Wnt/β-catenin signaling pathways.

Chronic inflammation contributes to amplifying and sustaining colonic tumorigenesis in *APC^Min/+^* mice [30, 31]. Pro-inflammatory cytokines, secreted by macrophages or tumor stroma, could excessively stimulate adenomatous polyp growth. In fact, pro-inflammatory cytokines were found to be involved in the progression of large adenomatous polyps, as well as overall polyp number [30]. In this study, we observed an elevation of IL-1β, IL-6, TNF-α and MCP-1 levels in adenomatous polyps and PGE_2_ and IL-6 in blood, which are considered to be indicators for rapid expansion of adenomatous polyps, in *APC^Min/+^* mouse model [30, 31]. Progression in tumor stage and size are associated with high levels of IL-1β, IL-6 and TNF-α [30, 31]. PGE_2_ is a potential stimulator that accelerates the proliferation of intestinal adenomatous cells through activation of prostaglandin receptor EP2-mediated cellular events [32]. In *APC^Min/+^* mice, the elevation of MCP-1, IL-1β, IL-6, TNF-α, and PGE_2_ was found to be associated with a rapid increase of adenomatous polyps at an earlier age [30, 31]. Further increase of these inflammatory cytokines with age was associated with increased size of adenomatous polyps [30, 31]. In addition to their stimulatory effect on adenomatous polyps, these cytokines were also found to provoke phosphorylated GSK3β and activate nuclear β-catenin, thereby further stimulating the expansion of adenomatous polyps [33]. Elevated levels of IL-1β, IL-6, TNF-α, MCP-1 and PGE_2_ are therefore accepted as major prognostic indicators for the progression of colon cancer. These cytokines are also valuable biomarkers for evaluating chemopreventive agents in *APC^Min/+^* mouse model [31]. Myricetin strongly reduced the levels of IL-1β, IL-6, TNF-α and MCP-1 in adenomatous polyps and PGE_2_ and IL-6 in blood. We thus suggest that myricetin might prevent the rapid increase of adenomatous polyps through the inhibition of chronic inflammation.

**Figure 8:**
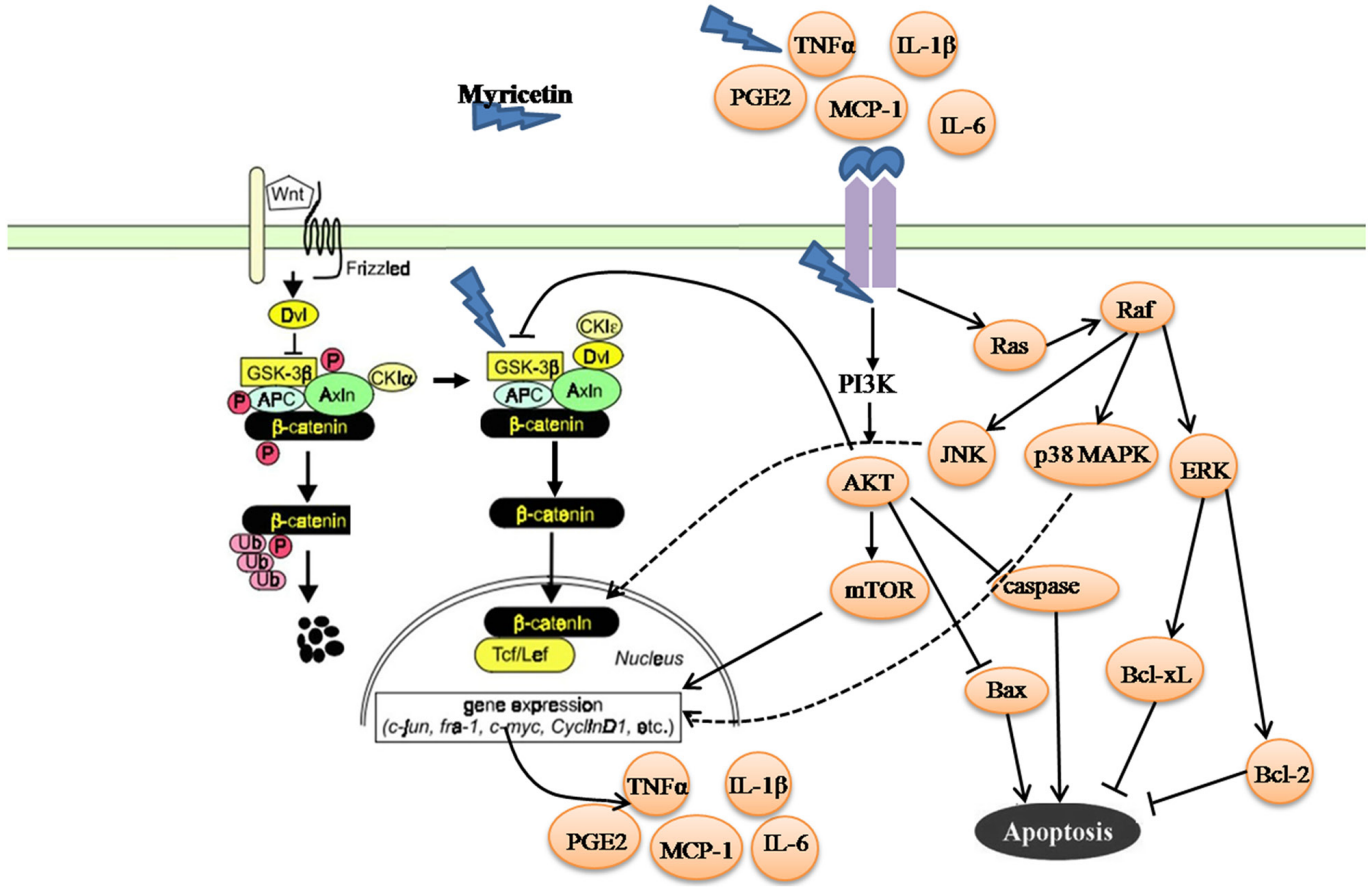
Summary for mechanisms of myricetin in the chemoprevention of intestinal tumorigenesis in *APC^Min/+^* mice *APC* as a canonical tumor suppressor forms a “destruction complex” with Axin/Axin2 and GSK-3β that promotes ubiquitination and proteasomal degradation of β-catenin. Loss of *APC* function leads to dysfunction of GSK-3β, and results in an accumulation of β-catenin, which translocates to nucleus and engages Tcf/Lef transcription factor complex to activate transcription of a large number of target genes, such as cyclin D1, PCNA, c-myc and CRDBP, etc. Myricetin exerts anti-proliferative, apoptotic and anti-inflammatory activities on the intestinal adenomatous polyps through multiple mechanisms including modulation of GSK-3β and β-catenin signaling pathways, and inhibition of p38 MAPK and Akt/mTOR signaling pathways.

The dysfunction of the p38 MAPK and Akt/mTOR signaling pathways might be the principal mechanism for controlling the status of cancer survival, apoptosis, proliferation, and differentiation in response to intracellular signals like deregulation of the Wnt/β-catenin pathway, and extracellular signals from the inflammatory or tumorigenic microenvironment [30, 34]. (i) Phosphorylated p38 MAPK and Akt/mTOR signaling pathways can disrupt the Wnt/β-catenin transcriptional activity [35]. In this study, myricetin inhibited the phosphorylated p38 MAPK/Akt/mTOR signaling pathways in the adenomatous polyps. Downregulation of p38 MAPK/Akt/mTOR signaling pathways could disrupt the nuclear functions of β-catenin, thereby blocking canonical Wnt signaling and consequently promoting the membranous translocation of β-catenin at adherens junctions [35]. (ii) The dysfunction of p38 MAPK/Akt/mTOR signaling pathways is frequently associated with decreased apoptosis [36]. The MAPK signaling pathway is composed of three subfamily members: ERK/MAPK, p38/MAPK and JNK [37]. ERK/MAPK is mainly involved in the response to mitogens, thereby eliciting cancer growth. p38/MAPK is frequently activated by environmental stress from chemotherapeutic agents [38]. Activation of p38/MAPK contributes to a decrease in apoptosis in the adenomatous polyps [39, 40]. JNK is preferentially activated in response to stress conditions. Activation of JNK is also associated with a decrease in apoptosis [41]. (iii) Extracellular signals, like cytokines and chemokines secreted in the inflammatory microenvironment, can activate the p38 MAPK and Akt/mTOR signaling pathways by binding to a chain of tyrosine kinases, which further stimulate transcription factors, thereby leading to cancer cell survival [42]. In the microenvironment of adenomatous polyps, myricetin reduced p38 MAPK and Akt/mTOR signaling. This could be the underlying mechanism of myricetin which results in reduced inflammation, less proliferation, and the induction of apoptosis in the adenomatous polyps (Figure 8).

In summary, our results indicate that myricetin inhibits the malignant progression of intestinal adenomatous polyps in *APC^Min/+^* mice. The effect of myricetin is associated with its multiple activities, including anti-inflammatory, anti-proliferative, and pro apoptotic effects. Long-term use of myricetin did not produce any significant adverse effects in mice, and we propose that myricetin could be used to reduce the risk of developing colon cancer.

## MATERIALS AND METHODS

### Myricein, animal model and chemoprevention study protocol

The animal protocol was approved by the Capital Medical University Institutional Animal Care and Use Committee. The permit number is AEEI-2014-101. The experiments were carried out in accordance with the approved guidelines. Myricetin (purity ≥ 96%) was purchased from Sigma Chemical Co (St. Louis, MO). The chemical structure 3,5,7,3′,4′,5′-hexahydroxyflavone was described elsewhere [43, 44]. Myricetin was dissolved in 5% sodium carboxymethyl cellulose (CMC-Na) (Sigma Chemical Co). C57BL/6J-*APC^Min/+^* male mice (J002020) were originally purchased from Jackson Laboratory (Bar Harbor, ME, USA) and breeding was continued at the Capital Medical University's Experimental Animal Lab. The room was maintained on a 12 h light:12 h dark cycle. Mice were crossed with wild-type C57BL/6 female mice to generate *APC^Min/+^* mice. *APC^Min/+^* mice carry a heterozygous mutation at codon 850 and develop approximately 30 intestinal polyps as described elsewhere [45]. A total of 18 (4 wk old) male mice were randomly divided into two groups. Mice were fed a standard mouse pellet diet, which contains 52% carbohydrate, 12% fat, 23% protein, 4% fiber, 6% ash, and 3% moisture [46]. After one week of acclimation, *APC^Min/+^* (6 weeks old) mice received either vehicle (CMC-Na 5%, v/v) or myricetin (100 mg/kg) by p.o. gavage daily (0.2 ml/10 g body weight). Administrations were performed five times per week for 12 consecutive weeks. Animals were weighed weekly and checked daily for any signs of illness. At 18 weeks of age, mice were anesthetized with ether and blood samples were collected by exsanguination from the inferior vein.

### Tissue processing and intestinal polyp scoring

Intestines were removed from each mouse and then sliced longitudinally, rinsed with saline and spread onto slides. The total number and size of intestinal polyps were examined under a dissecting microscope. Intestinal polyps were categorized by size (diameter) into 1-2 mm, 2-3 mm and >3 mm. After that, parts of the small and colonic intestines were snap-frozen in liquid nitrogen for western blot analysis. Other parts of the intestines were placed in 10% phosphate-buffered formalin for histopathology/immunohistochemical (IHC) analysis. Samples were dehydrated and embedded in paraffin as described elsewhere [47].

### Histopathology and immunohistochemical analysis

Sections (5 μm thick) were cut from the paraffin-embedded small and colonic intestinal polyps. For histopathological analysis, sections were stained with hematoxylin and eosin (H&E). For IHC analysis, sections were deparaffinized, processed for antigen retrieval and then endogenous peroxidase was blocked by incubation with 3% hydrogen peroxide in methanol. After that, the IHC assay was carried out in accordance with standard techniques. Primary antibodies included cyclin D1 (2922), β-catenin (9582) (Cell Signaling) and PCNA (ab29, Abcam). Secondary antibodies included anti-mouse IgG and anti-rabbit IgG (Santa Cruz). Positive staining with PCNA or cyclin D1 was defined as brown staining in the nuclei of adenomatous cells. The percentage of positive cells was calculated as described previously [23].

### In situ apoptosis detection by TUNEL staining

Apoptotic cells in the adenomatous polyps were identified by terminal deoxynucleotidyl transferase-mediated dUTP nick end labeling (TUNEL) staining using an *in situ* cell death detection kit (Roche, Germany). Apoptotic cells were identified as the brown DAB-positive cells and counted in five arbitrarily selected fields at ×400 magnification, together with the total number of cells in the field.

### Preparation of subcellular fractions of protein from intestinal polyps

Membrane protein of adenomatous cells was prepared with a NE-PER nuclear and cytoplasmic extraction kit, according to the manufacturer's instructions (Thermo Scientific). Cytoplasmic and nuclear proteins were extracted as previously described [48]. To prepare the adenomatous cell lysates, 3-5 polyps were dispersed in a cold cytoplasmic extraction reagent containing protease inhibitors. Lysates were centrifuged at 16000 g for 5 min at 4°C. The supernatant was collected as the cytoplasmic fraction and stored at −80°C. The insoluble pellets were re-suspended in hypertonic nuclear extract buffer containing protease inhibitors on ice for 10 min and then centrifuged at 16000 g for 10 min at 4°C. The supernatant was collected as the nuclear fraction and stored at −80°C for western blotting.

### Western blot analysis

The adenomatous polyps were excised from the intestines and pooled together based on treatment groups. To prepare lysates of adenomatous cells, 3-5 polyps were incubated with 80 μl RIPA lysis buffer at 4°C for 30 min. The supernatants were diluted and the concentration of protein was determined with a bicinchoninic acid kit (Pierce). The lysates (30 μg of protein per lane) were resolved by SDS-PAGE. Proteins were electro-transferred onto polyvinylidene fluoride (PVDF) membranes and detected with appropriate dilutions of primary antibodies. The primary antibodies included p-mTOR Ser^2448^ (5536), β-catenin (9582), p-β-catenin Ser^37^ (9561), cleaved caspase-3 (Asp175) (9664), IL-1β (12242), Bcl-xL (2764), cyclin D1 (2922), JNK (9252), p-JNK Thr^183^/Tyr^185^ (9251), Erk1/2 (9102), p-Erk Thr^202^/Tyr^204^ (9101), p38 MAPK (8690), p-p38 MAPK Thr^180^/Tyr^182^ (9211), Akt (4691), p-Akt Ser^473^ (4060, Cell Signaling), mTOR (sc-8319), Bax (sc-493, Santa Cruz), MCP-1 (GTX48813, GeneTex), GSK3β(ab32391), p-GSK3β Ser^9^ (ab75814), IL-6 (ab6672), TNF-α(ab6671), and β-actin (ab6276, Abcam). PVDF membranes were washed in 0.05% Tween-20/Tris-buffered saline and then incubated with horseradish peroxidase-conjugated secondary antibody. The bound antibodies were visualized using an enhanced chemiluminescence reagent (Millipore) and quantified by densitometry in a FluorChem FC3 image analyzer (Molecular Devices). Densitometric analyses of bands were normalized with β-actin functioning as a loading control.

### ELISA assay

The levels of serum IL-6 were quantified using commercially available sandwich ELISA kits, according to the manufacturer's protocol (EZMIL6, Meck Millipore). Concentration of PGE_2_ was quantified by an enzyme immunoassay (EIA) kit (ADI-900-001, Enzo Life Science). Briefly, diluted serum and PGE_2_ conjugates were added to a 96-well plate pre-coated with goat anti-mouse IgG for 2 h. The plate was washed with PBS to remove the unbound antibody-enzyme reagent. The substrate solution was added and then the intensity of color developed was read at a wavelength of 405 nm.

### Statistical analysis

Statistical analysis was done with SPSS/Win13.0 software (SPSS, Inc., Chicago, Illinois). Data were described as mean ± S.D. Comparisons between control and treatment groups were conducted by two-tailed Student's *t* tests. Multiple group comparisons were analyzed by one-way ANOVA and multiple between-group comparisons were performed using the S-N-K method. A p value less than 0.05 was considered statistically significant.
